# Role of mTOR inhibitor in the cellular and humoral immune response to a booster dose of SARS-CoV-2 mRNA-1273 vaccine in kidney transplant recipients

**DOI:** 10.3389/fimmu.2023.1111569

**Published:** 2023-02-02

**Authors:** Isabel Pérez-Flores, Ignacio Juarez, Arianne S. Aiffil Meneses, Ana Lopez-Gomez, Natividad Calvo Romero, Beatriz Rodriguez-Cubillo, María Angeles Moreno de la Higuera, Belen Peix-Jiménez, Raquel Gonzalez-Garcia, Elvira Baos-Muñoz, Ana Arribi Vilela, Manuel Gómez Del Moral, Eduardo Martínez-Naves, Ana Isabel Sanchez-Fructuoso

**Affiliations:** ^1^ Nephrology Department, Institute San Carlos for Medical Research (Instituto de Investigación Sanitaria del Hospital Clínico San Carlos (IdISSC), San Carlos Clinical University Hospital, Madrid, Spain; ^2^ Immunology Department, Complutense University School of Medicine, Madrid, Spain; ^3^ Microbiology Department, Institute San Carlos for Medical Research (Instituto de Investigación Sanitaria del Hospital Clínico San Carlos (IdISSC), San Carlos Clinical University Hospital, Madrid, Spain; ^4^ Department of Cell Biology, Complutense University School of Medicine, Madrid, Spain

**Keywords:** kidney transplantation, SARS-CoV-2 vaccine, immune response, COVID-19, mTOR

## Abstract

**Background:**

Immunocompromised patients have an increased risk of developing severe COVID disease, as well as a tendency to suboptimal responses to vaccines. The objective of this study was to evaluate the specific cellular and humoral adaptive immune responses of a cohort of kidney transplant recipients (KTR) after 3 doses of mRNA-1273 vaccine and to determinate the main factors involved.

**Methods:**

Prospective observational study in 221 KTR (149 non infected), 55 healthy volunteers (HV) and 23 dialysis patients (DP). We evaluated anti-spike (by quantitative chemiluminescence immunoassay) and anti-nucleocapsid IgG (ELISA), percentage of TCD4^+^ and TCD8^+^ lymphocytes producing IFNγ against S-protein by intracellular flow cytometry after Spike-specific 15-mer peptide stimulation and serum neutralizing activity (competitive ELISA) at baseline and after vaccination.

**Results:**

Among COVID-19 *naïve* KTR, 54.2% developed cellular and humoral response after the third dose (vs 100% in DP and 91.7% in HV), 18% only showed cell-mediated response, 22.2% exclusively antibody response and 5.6% none. A correlation of neutralizing activity with both the IgG titer (r=0.485, p<0.001) and the percentage of S-protein–specific IFNγ–producing CD8-T cells (r=0.198, p=0.049) was observed. Factors related to the humoral response in naïve KTR were: lymphocytes count pre-vaccination >1000/mm^3^ [4.68 (1.72-12.73, p=0.003], eGFR>30 mL/min [7.34(2.72-19.84), p<0.001], mTOR inhibitors [6.40 (1.37-29.86), p=0.018]. Infected KTR developed a stronger serologic response than naïve patients (96.8 vs 75.2%, p<0.001).

**Conclusions:**

KTR presented poor cellular and humoral immune responses following vaccination with mRNA-1273. The immunosuppression degree and kidney function of these patients play an important role, but the only modifiable factor with a high impact on humoral immunogenicity after a booster dose was an immunosuppressive therapy including a mTOR inhibitor. Clinical trials are required to confirm these results.

## Introduction

The coronavirus disease 2019 (COVID-19) pandemic has caused more than 6 million deaths worldwide (1), with immunocompromised individuals being particularly affected by severe conditions of this disease (2). Vaccination against severe acute respiratory syndrome coronavirus 2 (SARS-CoV-2) has been revealed as the most effective measure to control this pandemic, leading to a marked reduction in infections, hospital admissions and mortality (3). Individuals with COVID-19 who have undergone solid organ transplant (SOT) experience higher mortality and prolonged viral shedding compared with the general population (4–7). However, recipients of SOT were excluded from the initial licensing trials of these vaccines. Kidney transplant recipients (KTR), who undergo pharmacological immunosuppression as basic therapy to prevent transplant rejection, are at risk of a defective response to vaccination, as already occurs with other vaccines (8). In contrast to immunocompetent participants in vaccine trials (9), a low proportion of SOT recipients mount a positive antibody response to the second dose of SARS-CoV-2 messenger RNA (mRNA) vaccines. Studies have reported varying results in antibody response rates of approximately 5%–50% after two doses of mRNA vaccine in KTR (10–15). Due to this low response, an additional primary shot (third dose of mRNA COVID-19 vaccine for those receiving BNT162b2 or a booster dose of mRNA-1273) was recommended. Several published studies have reported the humoral immunogenicity of a three-dose vaccination schedule, but only a few have assessed the contribution of the cellular arm to vaccine-mediated protection (16–18). These results would allow us to determine if this regimen is sufficient to achieve a generalized response in these patients and would help us discern what type of immunosuppressive agents could cause a greater increase in the vaccine response.

In this study, we assessed antibody and cellular response after the third dose of mRNA-1273 vaccine in a cohort of KTR. We focused on the analysis of the cellular response and the neutralization capacity of the patients’ sera after the third dose.

## Methods

### Study design and sample collection

We performed a prospective study of a cohort of 221 KTR who received 3 doses of mRNA-1273 vaccine (Moderna-NIAID). Two cohorts with the same vaccination regimen, 55 healthy volunteers (HV) and 23 dialysis patients (DP), were also included as internal controls for the study. Patients and controls who became infected previously or during follow-up were excluded from the analysis of vaccine effectiveness.

We collected blood samples prior to vaccination (P0), 15 days (P1) and three months (P2) after the administration of the second dose, and 2 months after the third dose (P3).

The study was performed in accordance with the ethical standards as laid down in the Declarations of Helsinki and approved by the local ethics committee. Written informed consent was obtained from all subjects before the blood samples were taken.

### Patients

All KTR followed up in outpatient Kidney Transplantation Department between March 1 and April 15, 2021 and wanted to be vaccinated were included. All patients received the same kind of vaccine, mRNA-1273: a first and second dose (100 μg each dose) between April 20 and Maye 30, 2021 and third vaccine dose (100 µg) between September 20, and October 30, 2021. Inclusion criteria were: (1) being >18 years old, (2) History of kidney transplant for at least 6 months, and (3) Approval of informed consent to the study. As exclusion criteria; (1) having a history of malignancy, (2) SOT different from kidney, (3) primary immunodeficiency disease, (4) having a previous history of allergy to any inactivated vaccine, and (4) having an unexplained 37.5°C fever or any symptoms of infection.

### Controls

To characterize the impact of posttransplant immunosuppression on the ability of vaccination to elicit SARS-CoV-2–specific immunity, we used a control group of 78 non-immunocompromised, 55 healthy volunteers (HV) and 23 dialysis patients (DP). HV were healthcare workers who received mRNA-1273 and wanted to participate in the study. DP group was made up of 12 patient on hemodialysis and 11 on peritoneal dialysis who also received Moderna-NIAID vaccine. We obtained samples from HV at equivalent time intervals under the same conditions as KTR. For DP, only the sample corresponding to the third dose (P3) was obtained.

**Figure 1:**
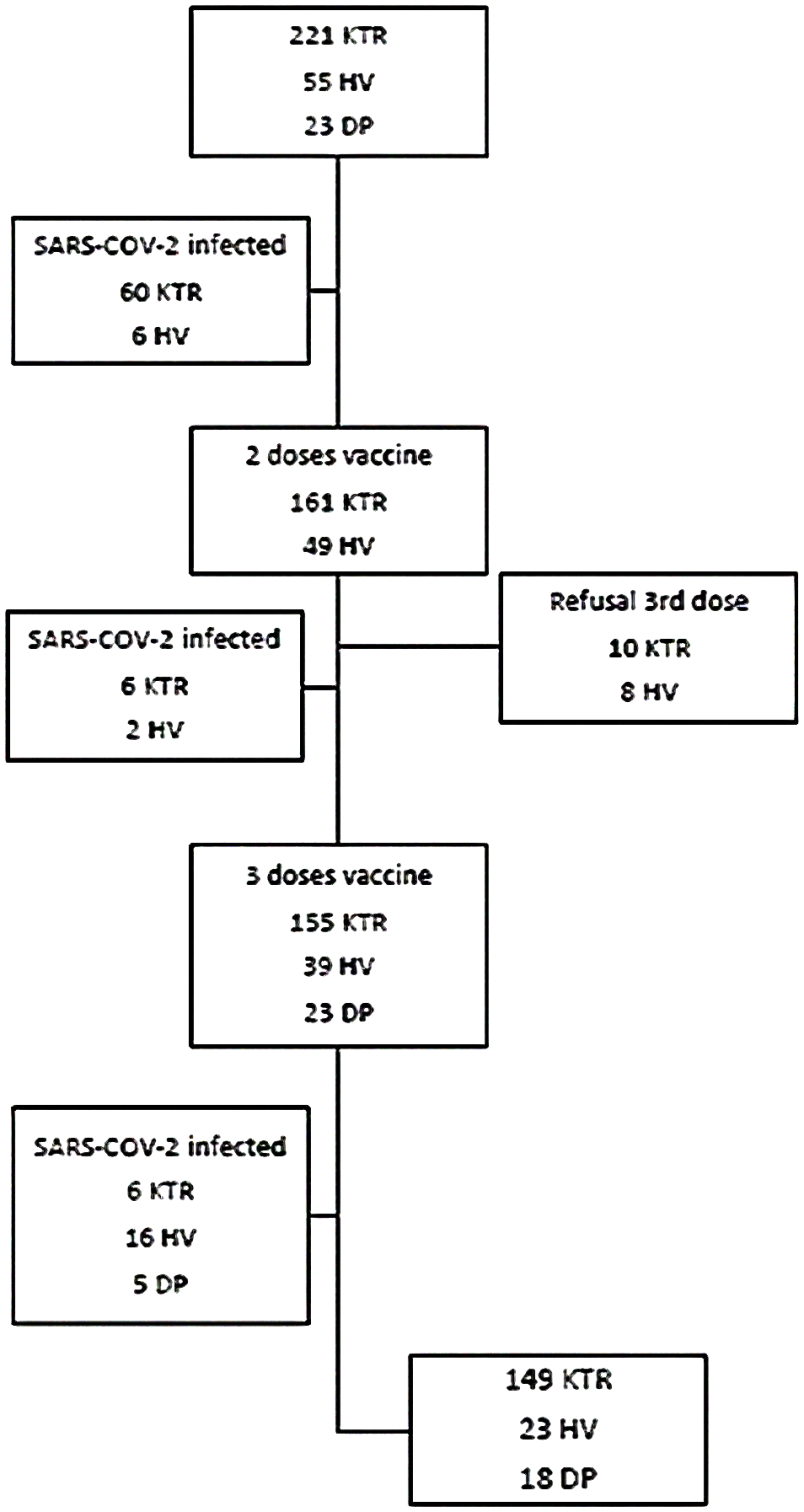
Study flowchart. Patients and controls who were infected or refused to the third dose were excluded from the analysis of vaccine effectiveness. KTR, Kidney transplant recipients; HV, Healthy volunteers; DP, Dialysis patients.

For both patients and controls, meeting any of the exclusion criteria throughout the study implied the individual’s exit from the study, as shown in the study flowchart (**Figure 1**).

### SARS-CoV-2 serology

Sera were obtained and stored at −80°C until use. Quantitative SARS‐CoV‐2 anti-spike (S) IgG test (SARS‐CoV‐2 IgG II Quant; Abbott Diagnostics) was performed in the Abbott Architect device in accordance with the manufacturer’s recommendations This antibody test is based on the principle of chemiluminescence microparticle immunoassay test. As the test gives data as AU/mL units, we applied a conversion factor in order to ease the comparison with other standardized serologic assessments, and quantitative results are given in BAU/mL (binding antibody units per mL, BAU/mL=AU/mL x 0.142). Samples with BAU/mL ≥7.1 are considered positive for SARS‐CoV‐2 IgG antibodies, the manufacturer-suggested thresholds (detection range, 2.8–16480 BAU/mL; positive agreement, 99.4%; negative agreement, 99.6%).

To determine which subjects had been infected prior to vaccination or in periods between sample collections, the presence of anti-Nucleoprotein (N) antibodies was tested by ELISA. Briefly, 96-well flat-bottom plates were coated with 2 μg/mL SARS-CoV-2 N-protein and 1:100 dilutions of the sera were incubated for 30 minutes at room temperature (RT), washed 5 times and detected with a goat anti-human IgG HRP-conjugated antibody (ThermoFisher Scientific). ELISA was developed with TMB and HCl and measured at 430 nm. To establish the cut-off of anti-N antibodies, we used the value of the mean plus twice the standard deviation (95% CI) of the absorbance value at 430nm of 8 pre-pandemic sera (PCR negative, anti-S IgG negative and with no COVID-19 compatible symptoms) per ELISA plate.

### Cell-mediated immunity

Peripheral blood mononuclear cells (PBMC) were obtained by density gradient isolation with Lymphocyte Separation Medium reagent (Corning Life Sciences). Cells were maintained in RPMI 1640 (Corning Life Sciences) supplemented with 10% FBS (Gibco), 100 mg/mL streptomycin (Gibco), and 100U/mL penicillin (Gibco). Cells were stimulated with 15-mer overlapping peptide-pool covering immunodominant domain surface Spike-protein (PepTivator^®^ SARS-CoV-2 Prot-S, Miltenyi Biotec) or with 10% DMSO for 6 hours at 37°C and 5% CO2 in the presence of Brefeldin A (Thermo-Scientific) during the last 4 hours of the assay. After stimulation, surface staining with anti-CD3-FITC (UCHT1), CD4-PE (OKT4) and CD8-PE/Cy7 (SK1) antibodies (Biolegend) was performed for 30 minutes at 4°C. After staining, they were fixed with 4% PFA for 30 min and permeabilized with 0.05% PBS Tween-20 (Thermo-Scientific) for 30 min at RT. Cells were stained with anti-IFNγ-APC antibody (B27) (Biolegend) for 30 min at RT. Staining was acquired on a FACSCalibur cytometer and analyzed with FlowJo v10 software (BD Life Sciences). Since the lower limit of detection for conventional flow cytometry techniques is ∼0.02 to 0.05%, we set 0.05% as the lower limit for considering the cellular response positive for both T-CD4 and T-CD8.

### ACE2-RBD neutralizing activity of the sera

To determine the ACE2-Spike (RBD) neutralizing activity of sera, we employed a competitive ELISA assay. 96 well-plate were coated with 2 μg/mL of recombinant RBD (Miltenyi Biotec) for 16h and incubated with 100 μL of the sera (1:25) for 1h at RT. After incubation, the plate was washed 5 times with 0.05% PBS-Tween and 0.5 μg/mL of recombinant ACE2-biotin (Miltenyi Biotec) was added at 37°C for 1 hour. After a washing step, a final incubation with 1μg/mL of streptavidin-HRP (Biolegend) was performed for 1 hour at 37°C. The interaction was retrieved with TMB and HCl (Thermo-Scientific) and absorbance is measured at 450 nm. To establish the maximum ACE2-RBD interaction, a pre-pandemic serum previously tested negative for anti-Spike antibody (0 BAU/mL) was used. From the maximum absorbance data, the decrease in signal of each serum with respect to the maximum was extrapolated to obtain the percentage neutralization data of each sample.

### Statistical analysis

Quantitative data were reported as mean and standard deviation (SD) or the median with interquartile range (IQR). Qualitative variables were expressed as absolute and relative frequencies.

Categorical variables were compared using the χ2 test. The Student’s *t* test or the Mann-Whitney U test was used for continuous variables. Repeated measures were compared with the Wilcoxon signed-rank test or the McNemar test, as appropriate. Correlations between continuous variables were evaluated with Spearman’s rho. Logistic regression served for assessment of factors related to immune response. All factors showing a univariate association with a *p*-value<0.100 were entered in the final multivariate model. All calculations were performed using GraphPad Prism version 8.0 (GraphPad) and SPSS version 25.0 (IBM). *P* < 0.05 (2-sided) was considered statistically significant.

## Results

### Study population

Antibody response to the vaccine was determined in a total of 221 KTR and cellular immunity in 213 of them (viable T cells could not be obtained in 8 samples). Fifty-two patients (23.5%) had a history of prior COVID-19 diagnosed 12 months ago, 11 of them met criteria for severe COVID-19. Eight KTR became infected between the first and second dose of vaccine, six patients after the second dose and six after the third (9%), all of them with mild symptoms (**Figure 1**). The main clinical, analytic, and demographic characteristics of this cohort, *naïve* and infected KTR, are described in **Table 1S**. There were not significant differences in laboratory parameters during follow up in both groups (data not shown).

Non transplant control group made up of HV were younger than KTR [30( ± 8) vs 57( ± 15) years, p<0.001], while DP were similar in age [56( ± 13) years]. The incidence of SARS-CoV-2 infection, assessed by PCR or positivity against N-protein in HV was 11% (6/55) at baseline, 4% (2/49) after the second dose and 34% (16/47) after the third dose and 5/23 in DP after the third dose (**Figure 1**). Patients and controls who were infected were also analyzed to see if there were differences between them, but they were excluded from the analysis of vaccine effectiveness.

### SARS-CoV-2–specific cell-mediated immunity and correlation with total and neutralizing titers against the S-protein after the third vaccine dose

**Figure 2:**
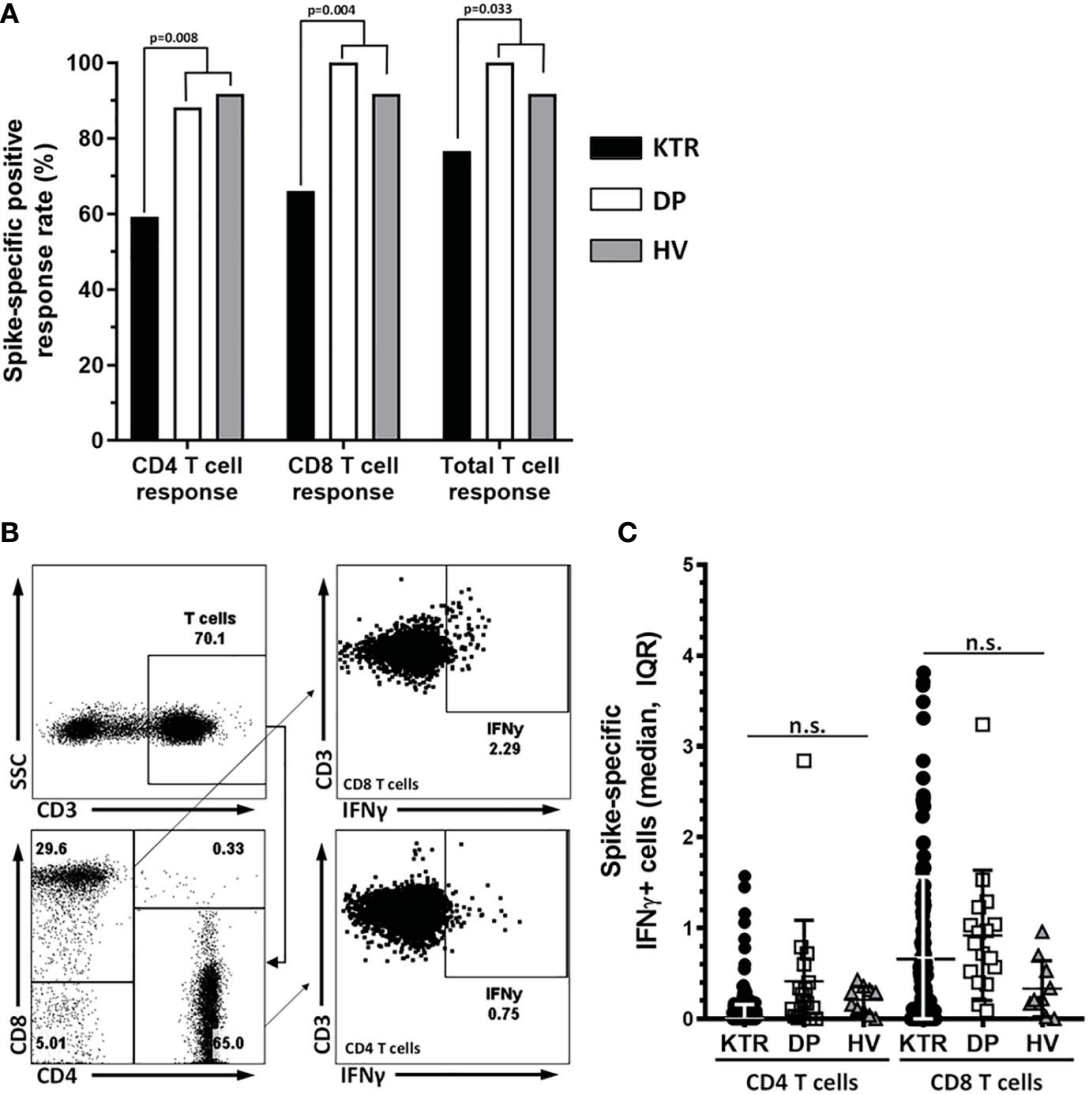
Cellular response rate after the third dose of vaccine. **(A)** Cellular response rate in CD4, CD8 and total T lymphocytes. A spike-specific response of IFNγ producing T-cells (%) >0.05 was consider as positive. The Y axis represents the response rate in CD4, CD8 and total T lymphocytes, for each subgroup of patients. **(B)** DotPlots show the gatting strategy to analyze the response to interferon gamma after stimulation of PBMC with the protein S peptide pool in CD8 and CD4 T cells. **(C)** Graph show Spike-specific IFNγ+ cells in CD4 and CD8 T cells median and IQR of KTR, DP and HV after three doses of vaccination. The median percentage of IFNy-producing CD4-T cells were 0.17% (0-0.90 IQR) in KTR, 0.20% (0.07-0.50 IQR) in DP and 0.29% (0.09-0.35 IQR) in HV (p=0.820). In the case of CD8-T cell response the median percentages were: 0.41% (0-1.06 IQR) in KTR, 0.83% (0.46-1.13 IQR) in DP and 0.23% (0.17-0.65 IQR) in HV (p=0.054). n.s., non significant.

The proportion of positive S-protein–specific cell-mediated response after the third dose were lower in KTR compared to DP and HV ones: 59.3% of KTR showed reactive CD4-T cells vs 88.2% of HV and 100% of DP (p=0.008); 66% of KTR showed reactive CD8-T cells vs 100% in DP and 91.7% in HV (p=0.004). CD4 or CD8 reactivity was present in 76.7% of KTR vs 100% in DP and 91.7% in VH, p=0.033 (**Figures 2A, B**). There were not significant differences in the intensity of cellular response between groups (**Figure 2C**).

Analyzing humoral and cellular response in COVID-19 naïve KTR, 54.2% (78/144) had both responses, 18% (26/144) mounted cell-mediated responses without IgG response, 22.2% (32/144) only developed antibody response and 5.6% (8/144) did not develop any response.

In COVID-19 *naïve* KTR with positive cellular response, there was a significant correlation between the percentage of S-protein–specific IFNγ–producing CD8-T cells and total anti-S IgG titers after the third dose (P3: *r*=0.210, p=0.043) and between the percentage of S-protein–specific IFNγ–producing CD8-T cells and neutralizing titers against the S-protein (P3: *r*=0.307, p=0.004). No correlation was found between CD4-T cells and humoral response. In the univariate analysis we did not find any parameter that could predict the cellular response (**Table 1**).

**Table 1:** Comparison of clinical characteristics between KTR who did or did not mount cellular and humoral response two months after 3 doses of mRNA-1273 (P3).

	SARS-CoV-2 specific humoral response			SARS-CoV-2 specific cellular response			
	Responders N=112	Non responders N=37	p	Responders N=109		Non responders N=35	p
*Gender (male), N (%)*	68 (60.7)	21 (56.8)	0.670	63 (57.8)		23 (65.7)	0.406
Age of recipient							
* years (mean, SD)*	58.6 (15.2)	62.0 (13.2)	0.670	59.0 (15.2)		60.8 (13.6)	0.698
* >60 y, N (%)*	56 (50.0)	25 (67.6)	0.063	58 (53.2)		20 (57.1)	0.685
*Diabetes, N (%)*	39 (34.9)	14 (37.8)	0.724	40 (36.7)		12 (34.2)	0.738
Time since transplantation							
* years (median, IQR)*	9.9 (5.6-16.6)	9.2 (3.6-13.7)	0.477	9.6 (5.3-16.7)		9.5 (4.8-12.4)	1
* <5 years, N (%)*	24 (21.4)	11 (29.7)	0.302	25 (22.9)		9 (25.7)	0.736
*Previous Transplant*, *N (%)*	14 (12.5)	4 (10.8)	0.895	10 (9.1)		7 (20)	0.053
Immunosuppressive drug, N (%)							
* CNI*	86 (76.8)	34 (91.9)	0.044	88 (80.7)		28 (80)	0.924
* MPA*	87 (77.6)	34 (91.9)	0.053	86 (78.9)		29 (85.3)	0.412
* mTORi*	35 (31.2)	2 (5.4)	0.002	27 (24.8)		10 (28.6)	0.654
* Thymoglobulin*	57 (50.9)	27 (73)	0.016	57 (52.2)		23 (65.7)	0.134
*Immunosuppressive protocol, N (%)*			0.004				0.715
* MPA+CNI*	73 (65.1)	35 (94.5)	0.001	80 (73.3)		25 (71.4)	0.716
* MPA+mTORi*	16 (14.2)	0 (0)	0.017	10 (9.1)		5 (14.2)	0.418
* mTORi+CNI*	23 (20.5)	2 (5.4)	0.046	19 (17.4)		5 (14.2)	0.804
* eGFR (mL/min/1.73 m^2^), median (IQR)*	49.5 (38.1-71.4)	30.1 (21.3-41.4)	0.001	46.1 (30.0-64.4)		45.4 (36.3-63.0)	0.991
*Stages CKD, N (%)*			0.001				0.288
* >60 mL/min/1.73 m^2^*	43 (38.4)	1 (2.7)		33 (30.3)		10 (28.6)	
* 30-60*	55 (49.1)	17 (45.9)		48 (44.0)		20 (57.1)	
* <30*	14 (12.5)	19 (51.4)		28 (25.7)		5 (14.3)	
Cells count before vaccination, 1x10^3^/mm^3^, median (IQR)							
* Lymphocyte*	1.6 (1.1-2.0)	1.0 (0.7-1.6)	0.008	1.5 (1.0-1.8)		1.5 (1.1-2.3)	0.525
* CD4^+^ T cells*	5.4 (3.9-7.7)	4.1 (2.7-6.6)	0.807	5.1 (3.5-8.9)		5.4 (3.5-7.6)	0.638
* CD8^+^ T cells*	4.5 (3.0-7.4)	3.8 (1.8-7.6)	0.068	3.9 (2.6-6.9)		6.1 (3.6-7.1)	1
Lymphocyte							
*>1x10^3^/mm3, N (%)*	92 (82.1)	20 (54.1)	0.001	79 (72.5)		29 (82.8)	0.129
Serum Immunoglobulins levels, mg/dL, median *(IQR)*							
* IgG*	1050 (877-1275)	939 (750-1125)	0.112 0.092	1020 (843-1245)		1020 (890-1130)	0.845
* IgA*	227(137-292)	148(111-281)	0.229	221(122-294)		165(131-257)	0.435
* IgM*	86(60-120)	72.5(35-116)		83(52-121)		79(48-105)	0.922

CNI, Calcineurin inhibitor; MPA, mycophenolic acid; mTORi, mammalian Target Of Rapamicin inhibitor; eGFR (CKD-EPI), estimated glomerular filtration rate; IQR, interquartile rate; SD, Standard deviation.

### SARS-CoV-2 IgG antibody response and serum ACE2-RBD neutralizing activity. Strong impact of immunosuppressive therapy

**Figure 3:**
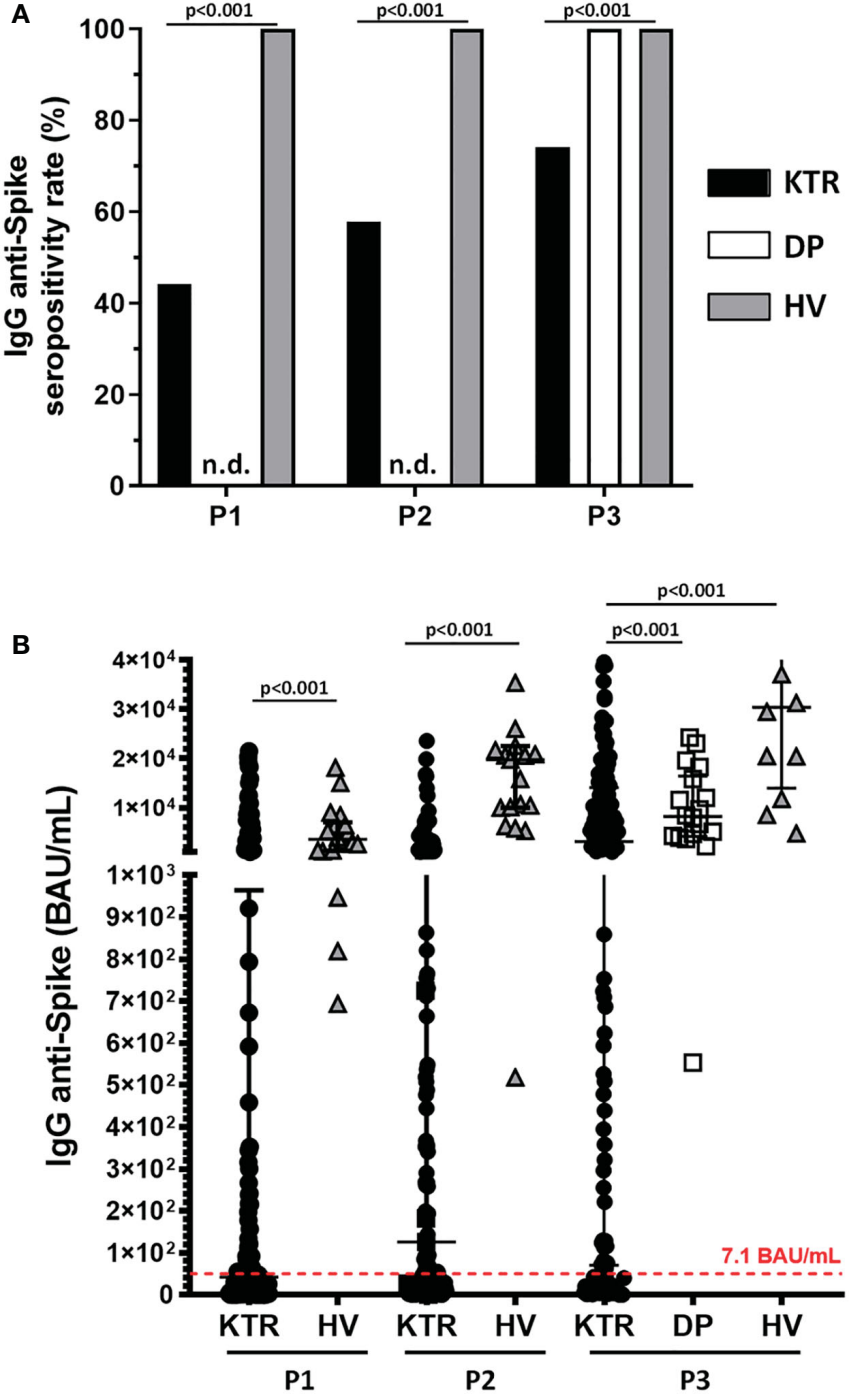
Kidney transplant recipients have lower humoral response rates and lower antibody titers after vaccination. **(A)** Seropositivity rate after vaccination in patients (KTR) and control groups (dialysis, PD; and healthy volunteers, HV) for each sample collection after vaccine administration. A threshold of >7.1 BAU/mL was used to consider seropositivity. **(B)** Antibody levels (BAU/mL) for each sampling and group after vaccine administration. Serum anti-spike IgG titers were higher in HV compared to KTR after second dose: 1782.8 (719.2-2519.2 IQR) vs 4.4 (0.3-135.6 IQR) BAU/mL at P1 (p<0.001), 394.3 (179.1-850.6 IQR) vs 18.3 (1.2-140.3 IQR) BAU/mL at P2 (p<0.001) and after the third dose: 335.5 (7.1-1415.3 IQR) BAU/mL in KTR, 908.9 (457.8-1872.8 IQR) BAU/mL in DP, 3058.9(1405.4-4366.1 IQR) BAU/mL in HV, p<0.001 Data were expressed as median and IQR, and p-values for each Mann-Whitney U test. n.d., non-determined.

</u>Using the manufacturer-suggested thresholds, the rate of IgG seropositivity in COVID-19 *naïve* KTR was 44.1% (74/161) at 15 days (P1) and 58% (90/155) at 3 months (P2) after the second dose. This rate increased to 76.5% (114/149) at 2 months after the booster dose (P3). Significant differences in the humoral response were observed with the control group: 100% positivity in the three points in HV and 100% in DP after the third dose (p<0.001) (**Figure 3A**). Likewise, the serum anti-spiked IgG titers were higher in HV compared to KTR after the second (p<0.001) and the third dose (p<0.001) (**Figure 3B**). The evolution of IgG titers also differed between the control population and the KTRs (p<0.001). Some KTR showed a delay in antibody production as seen on (**Supplementary Figure 1**).

The factors associated with humoral immunogenicity in KTR are describe in **Tables 1**, **2**.

**Table 2:** Factors related to antibody respond after second and third dose of SARS-CoV-2 mRNA-1273 vaccine in COVID-19 naïve KTR.

	UNIVARIATE ANALYSIS OR (95% ci), p	MULTIVARiate analysis OR (95% ci), p
P1 (N=161)		
**Age >60 y**	0.69 (0.36-1.32), 0.269	
**Gender**	1.02 (0.52-1.97), 0.953	
**Diabetes**	0.64 (0.31-1.30), 0.222	
**Time since transplantation >5 y**	0.60 (0.27-1.32), 0.203	
**Previous transplant**	1.55 (0.56-4.30), 0.390	
**mTOR inhibitor**	7.78 (3.37-17.97),<0.001	
**MPA**	0.06 (0.02-0.18), <0.001	0.05 (0.01-0.20), <0.001
**CNI**	0.32 (0.14-0.76), 0.008	0.17 (0.06-0.50), 0.001
**eGFR pre-vaccination>30 ml/min/1.73 m^2^**	2.48 (1.06-5.81), 0.032	6.08 (1.84-20.06), 0.003
**Lymphocyte count pre-vaccination >1x10^3^/mm^3^**	2.50 (1.10-5.68), 0.025	3.47 (1.24-9.68), 0.017
**Thymoglobulin**	0.58 (0.29-1.14), 0.114	
P2 (N=155)		
**Age >60 y**	0.49 (0.26-0.91), 0.024	
**Gender**	1.22 (0.46-3.68), 0.521	
**Diabetes**	0.51 (0.26-0.98), 0.044	
**Previous transplant**	1.31 (0.50-4.18), 0.607	
**Time since transplantation >5 y**	0.50 (0.24-1.00), 0.050	
**mTOR inhibitor**	4.45 (1.93-10.24), <0.001	
**MPA**	0.22 (0.08-0.56), 0.001	0.10 (0.03-0.31), <0.001
**CNI**	0.58 (0.26-1.31), 0.191	
**eGFR pre-vaccination>30 ml/min/1.73 m^2^**	3.33 (1.53-7.23), 0.002	4.78 (1.67-13.65), 0.003
**Lymphocyte count pre-vaccination >10^3^/mm^3^**	3.78 (1.78-8.02), <0.001	5.03 (1.93-13.10), 0.001
**thymoglobulin**	0.36 (0.18-0.72), 0.003	0.34 (0.15-0.74), 0.007
p3 (n=149)		
**Age >60 y**	0.48 (0.22-1.04), 0.063	
**Gender**	1.17 (0.55-2.50), 0.670	
**Diabetes**	0.86 (0.39-1.92), 0.724	
**Previous transplant**	1.08 (0.32-3.56), 0.895	
**Time since transplantation >5 y**	0.64 (0.27-1.48), 0.302	
**mTOR inhibitor**	7.95 (1.81-34.91), 0.002	6.40 (1.37-29.86), 0.018
**MPA**	0.30 (0.08-1.07), 0.053	
**CNI**	0.29 (0.08-1.02), 0.044	
**eGFR pre-vaccination>30 mL/min/1.73m^2^**	7.38 (3.14-17.35), <0.001	7.34 (2.72-19.84), <0.001
**Lymphocyte count pre-vaccination >10^3^/mm^+3^**	4.11 (1.82-9.28), <0.001	4.68 (1.72-12.73), 0.003
**Thymoglobulin**	0.35 (0.15-0.84), 0.016	

P1: 15 days after second dose; P2: three months after second dose; P3: two months after third dose; MPA,: mycophenolic acid; CNI, calcineurin inhibitor; mTOR, mammalian Target Of Rapamycin; eGFR, estimated glomerular filtration rate.

Univariate and multivariate regression model (adjusted to age, gender and time since transplantation).

Antibody titers were correlated to kidney function measured by estimated glomerular filtration rate (eGFR) (P1: *r*=0.333, P2: *r*=0.482, P3: *r*=0.550, p<0.001). KTR with better renal function pre-vaccination (eGFR>30 mL/min/1.73 m^2^) achieved a higher humoral response rate than those with lower renal function after the second and third dose (P1: 48.3 vs 27.3%, p=0.032; P2: 63.5 vs 34.4%, p=0.003; P3: 84.5 vs 42.4%, p<0.001).

On the other hand, those patients with **lymphocyte count** greater than 1000/mm^3^ were almost five times more likely to respond after the second [P2: OR 4.46 (1.64-12.13 CI), p=0.003] and third dose [P3: OR 4.68 (1.72-12.73 CI), p=0.003]. A correlation between lymphocyte count and antibody titers were also detected (P1: *r*=0.185, p=0.017; P2: *r*=0.263, p=0.001, P3: *r*=0.284, p<0.001).

**Figure 4:**
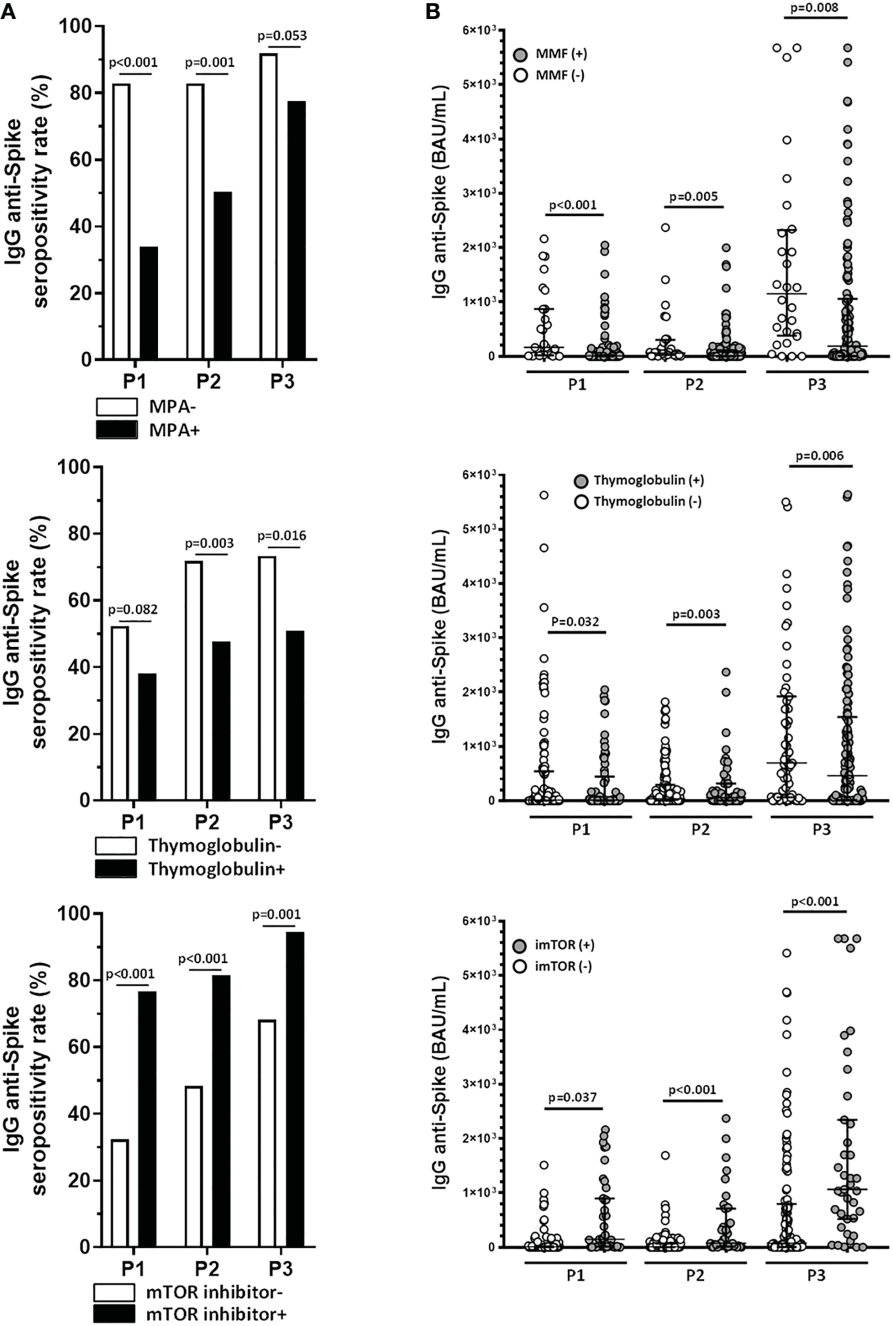
Treatment with mycophenolate and thymoglobulin reduces the efficacy of the humoral response in kidney transplant recipients, whereas therapy with mTOR inhibitors reverses the negative effect of immunosuppressive therapy in these patients. **(A)** Humoral response rates comparing mycophenolate (MPA), thymoglobulin and mTOR inhibitor therapies. **(B)** Levels of anti-Spike IgG antibodies comparing therapies with mycophenolate (MPA), thymoglobulin and mTOR inhibitors. Patients under MPA had lower IgG anti-spike titers than those without it in all points (P1: 2.6 (0.1-19.9) vs 149.4 (12.4-966.3) BAU/mL, p<0.001; P2: 7.1 (0.7-109.9) vs 50.1 (9.5-300.5) BAU/mL, p=0.005; P3: 104.2 (5.3-1051.5) vs 737.5 (213.1-2183.5) BAU/mL, p=0.008). The same behavior was performed in KTR who received thymoglobulin (P1: 4.5 (0.1-135.5) vs 13.8 (0.7-759.4) BAU/mL, p=0.032, P2: 5.8 (0.6-68.0) vs 46.9 (4.3-317.2) BAU/mL, p=0.003, P3: 56.7 (2.3-877.8) vs 1378.1 (53.0-1919.3) BAU/mL, p=0.006). In patients treated with mTORi, antibody titers were higher vs non-treated (P1: 162.7 (9.2-1093.4) vs 2.6 (0.1-20.3) BAU/mL, p<0.001; P2: 145.6 (12.6-709.4) vs 6.0 (0.7-70.4) BAU/mL, p<0.001; P3: 1036.2 (366.4-2270.0) vs 70.3 (3.4-761.7) BAU/mL, p<0.001). Graphs include data for each sampling after vaccination and p-values for each Mann-Whitney U test.

Finally, the immunosuppressive therapy also had an influence on the antibody as can be seen in **Tables 1**, **2**. Non-responders *after the second dose* were more frequently under MPA (P1: 82.9 vs 33.9%, p<0.001; P2: 82.9 vs 50.4%, p=0.001; P3: 91.9 vs 77.5%, p=0.053) or had previously received thymoglobulin (P1: 52.3 vs 38.2%, p=0.082; P2: 71.9 vs 47.7%, p=0.003; P3: 74.3 vs 50.9%, p=0.016), whereas responders *after the booster dose* were more likely to receive mTOR inhibitor (mTORi) (P1: 76.7 vs 32.3%, p<0.001; P2: 81.4 vs 48.3%, p<0.001; P3: 94.6 vs 68.2%, p=0.001) (**Figure 4A**). This protective effect of mTORi was maintained regardless of combination of drugs (**Table 1**).

Moreover, differences are observed in the quantitative response in such a way that patients under MPA had lower IgG anti-spike titers than those without it in all points. In the case of use of mTORi, antibody titers were higher in patients who received it than the others (**Figure 4B**).

In the multiple logistic regression, MPA (p<0.001) and thymoglobulin (p=0.007) use were associated with lack of response to vaccine after second dose. However, the only immunosuppressor with significant association with the response after the third dose was mTORi (p=0.018), the most common drug among responders (**Table 2**).

**Figure 5:**
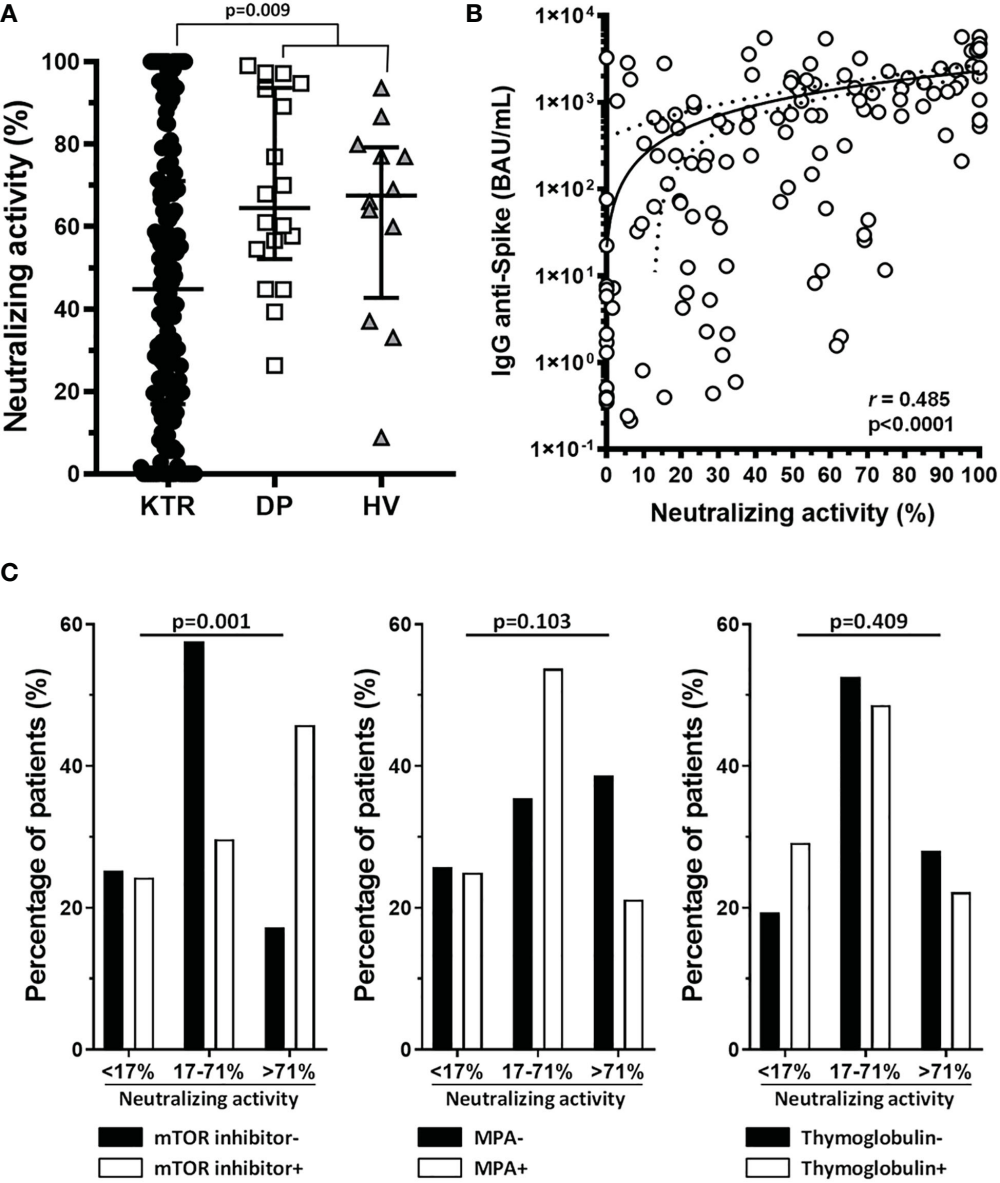
Sera from renal transplant recipient patients show lower RBD-ACE2 binding neutralizing activity than control groups, which correlates with antibody levels, and mTOR inhibitors treatment enhance neutralizing activity of sera of these patients. **(A)** Graph shows the neutralizing activity of patient sera at 1:25 dilution in patients (KTR) and control groups (DP and HV), with median and IQR, and p-value for Mann-Whitney U test. **(B)** Graph shows the correlation of neutralizing activity with antibody levels calculated with Spearman’s Rho. **(C)** Graphs show the percentage of patients with a range of neutralizing activity (divided in three percentiles, according to p25 and p75, <17%, 17%-<71% and >71%) for mTOR inhibitor, MMF and thymoglobulin treatment after the third dose of vaccination.

Regarding serum neutralizing activity against the S-protein after the booster dose of vaccine, low percentage of neutralizing activity were found in KTR compared to those of DP and HV: 44.8 (16.9-71) vs 64.4 (52-93.6) vs 67.5% (42.7-79.1), respectively (p=0.009) (**Figure 5A**). Besides of correlation between neutralizing titers against the S-protein and T-cell response, as we described above, there were an association between anti-S neutralizing activity and total IgG titers (*r*=0.485, p<0.0001) (**Figure 5B**).

As with the IgG titer, we observed a relationship between neutralizing activity after the third dose, categorized according to p25 and p75, and mTORi; such that patients treated with this drug were more likely to develop greater neutralizing activity after vaccination (p=0.001). There were no differences in neutralizing activity when we analyzed MPA or thymoglobulin treatment (**Figure 5C**).

### SARS-CoV-2 infected patients

**Figure 6:**
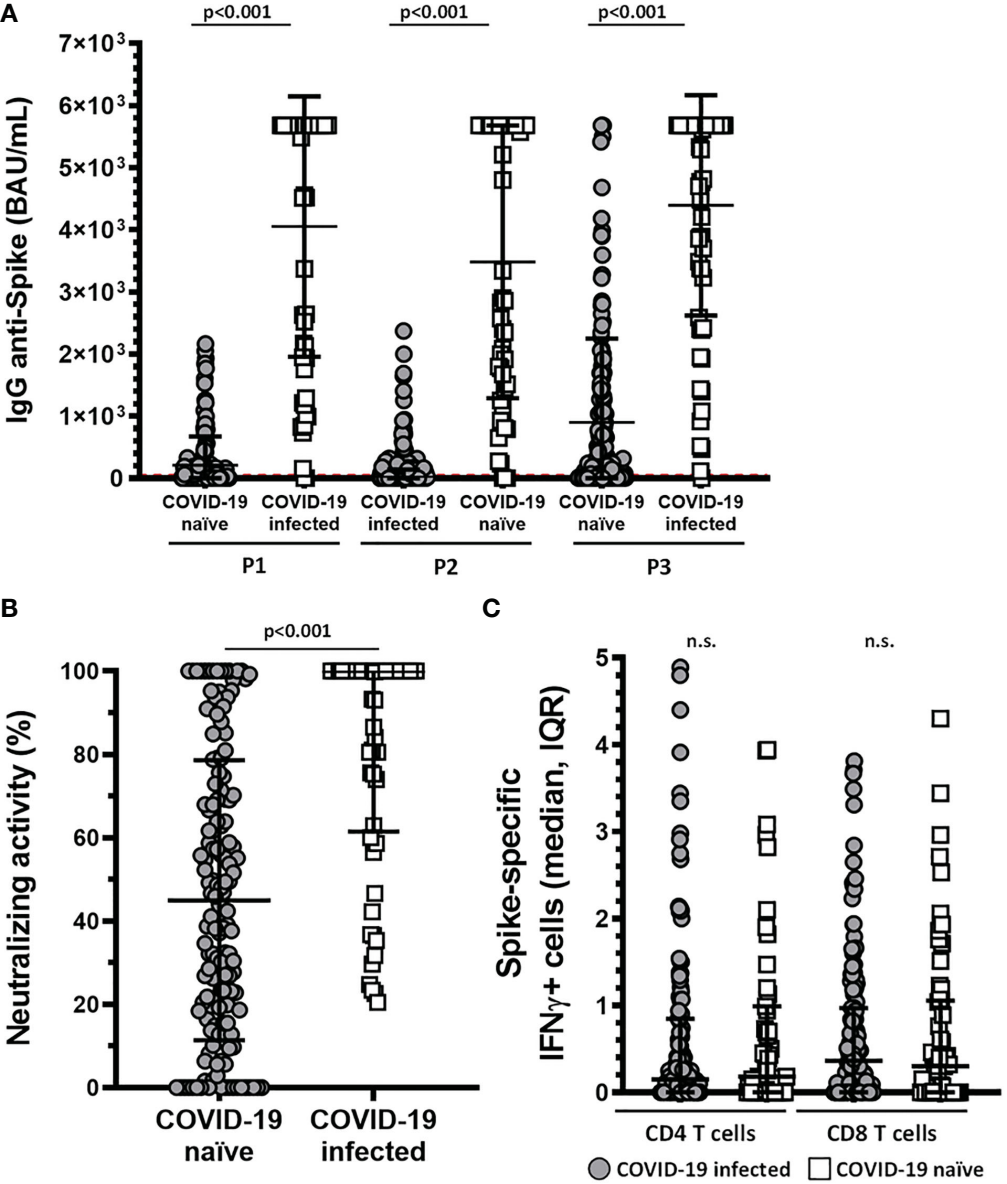
Patients affected by COVID-19 have higher levels of anti-Spike IgG antibodies and higher neutralizing activity. **(A)** Levels of anti-Spike IgG antibody (BAU/mL) in COVID-19 affected and unaffected patients for each of the post-vaccination samples (p<0.001). **(B)** Neutralizing activity of sera after the third dose in COVID-19 affected and unaffected patients. Neutralizing activity was 78.9 in SARS-CoV-2 infected vs 43.4% (p<0.001) in SARS-CoV-2 *naïve* KTR. Both graphs show median and IQR, an p-value for each Mann-Whitney U test. **(C)** Graph show IFNγ+ cells in COVID-19 naïve and COVID-19 infected individuals in CD8 and CD4 T cells. Non-significant differences were found between both groups. n.s., non significant.

Finally, we compared SARS-CoV-2–specific immunity elicited by mRNA-based vaccine between infected and non-infected patients. The rate of antibody response was higher in infected than COVID-19 *naïve* KTR; the seropositivity anti-S IgG was 96.8 vs 75.2% (p<0.001) and neutralizing activity, 98.2 vs 80.9% (p<0.001) (**Figure 6B**). Significant differences were also detected in the intensity of this humoral response between both groups. IgG titers were 5680.0 (3460.0-7524.7) versus 335.5 (7.1-1415.3) BAU/mL (p<0.001) (**Figure 6A**) and neutralizing activity in 78.9 vs 43.4% (p<0.001) in SARS-CoV-2 infected vs *naïve* KTR, respectively (**Figure 6B**).

Nevertheless, we did observe no differences in the cellular response among infected and *naïve* KTR: reactive CD4-T cells, 64.4 vs 58.3% (p=0.422); reactive CD8-T cells, 67.8 vs 64.6% (p=0.662) (**Figure 6C**).

## Discussion

In this study, we analyzed SARS-CoV-2–specific cell-mediated and humoral immunities following two and booster doses of mRNA-1273 vaccine. KTR showed a marked reduction in the response rate, with a link between different types of immunosuppressive therapy.

As for cellular response, we did not find a correlation between the different clinical or treatment variants of the patients, although the transplanted cohort showed a lower response rate, both in CD8 and CD4 T cells, compared to control group. These results coincide with those of other studies in cohorts of patients with pharmacological immunosuppression, such as hematological cancer (19), as well as patients with various types of immunodeficiencies (20, 21). These studies also found no correlation between T-cell response and the clinical characteristics of the patients. This fact could be due to the high heterogeneity of the response against specific pools of the SARS-CoV-2 Spike protein (22).

In contrast to studies evaluating cellular response in PBMC pool or whole blood, our study has allowed independent study of CD4 and CD8 T response, identifying a higher response rate in CD8-T cells from patients undergoing KTR.

With regard to humoral response, KTR had a lower IgG response rate compared to the control group in each period, and a delay in the antibody production. In fact, some patients with low titers at day 15 after the administration of the second dose, raised antibody titers 3 months after this dose, in opposition to the downward curve observed in the general population (23).

We observed that the initial immunization schedule did not generate an adequate IgG response in KTR, and the third dose was not sufficient to rescue all non-responders, similar to other immunosuppressed populations (24). These patients could require several booster doses and seasonal vaccination patterns, as is already the case for other types of infectious diseases, like influenza vaccination (25).

Our data showed that reduced renal function decreased the likelihood of achieving seroprotection both after the second and third doses, as has been described in H1N1 vaccination (26). The mechanisms are still not very clear, since significant humoral response is observed in 100% of DP in this and other studies (27).

Also, we found an influence of lymphocyte count and lymphocyte depletion treatment, even when it was administrated several years earlier. Some studies have reported that lymphopenia is associated with infectious complications in cancer (28–30) and that there is an age-dependent decline in the capacity of the adult immune system to regenerate lymphocytes after thymoglobulin administration (31).

Interestingly, the prospective design and sample size and homogeneity of our cohort, which received the same vaccination type and schedule, also allowed us to identify relevant correlations with patient therapies. The immunosuppressive treatment had a different impact depending on whether we analyzed the response to second or booster dose. With the initial vaccination schedule (two doses), patients treated with MPA showed a pronounced decrease in IgG response compared to the rest of patients. Conversely, a higher probability of positive humoral response following the second dose was observed in those with mTORi in the univariate analysis. The relationship between mTORi and a better immune response were recently described by Netti et al (32). Nevertheless, this beneficial effect of mTORi after two doses of vaccine was no maintained in the adjusted model in our cohort, findings also noted by Bae (33). Several authors found similar results with MPA and mTORi in different groups of patients who received two doses (33–37), even proposing the temporary suspension of treatment during the vaccination process (38).

But we go further and performed an analysis of immune response after the third or booster dose and different results were observed. There was no association between humoral response and MPA, and mTORi was the only treatment that showed an independent association with immunogenicity following the third dose. We hypothesized that the negative impact of MPA was diluted after booster dose, perhaps due to greater antigenic exposure, similar to what happened in the case of patients who have undergone COVID-19 in any of the study periods. The mTORi-treated patients with three doses of vaccine showed a quantitative and qualitative humoral immune response similar to controls, with high response rates. Several studies found that mTORi can enhance the formation and differentiation of memory CD8 T cells in anti-tumor vaccines and in immunization against viruses and parasites (39–42). It has been suggested that mTOR blockade effectively potentiated antigen-specific T-cell and B-cell responses induced by HBV vaccines (43).

Finally, we found that neutralization capacity after the third dose is clearly linked to anti-S IgG antibody titers, as had already been described (44). This is especially relevant in KTR, as many of them generate response after vaccination, but with low titers, which may lead to an increased risk of infection and complications. As with antibody titers, treatments affected the neutralizing capacity of these sera. Patients treated with mTORi had a greater neutralization capacity, as they achieved higher IgG titers, suggesting a more efficient post-vaccination response. This data may lead to consider the use of this therapy as an adjuvant for the response to new booster doses of mRNA vaccines.

Regarding the limitations of this study, all the assays were performed on the wildtype strain of SARS-CoV-2. In addition, the administration of a fourth dose to immunosuppressed patients has been standardized, so we have extended this study to verify the effect of the fourth dose.

In conclusion, this study shows that KTR have a lower response after to doses of mRNA-1273 vaccination, especially accentuated in those treated with MPA or thymoglobulin. Based on these observations, it can be assumed that COVID-19 still presents a major risk for vaccinated KTR. However, it is possible to rescue patients with the third dose and mTORi therapy could be a potential adjuvant therapy to improve the response to booster doses in this high-risk population.

## Data availability statement

The original contributions presented in the study are included in the article/**Supplementary Material**. Further inquiries can be directed to the corresponding author.

## Ethics statement

The studies involving human participants were reviewed and approved by Ethics Committee of Hospital Clínico San Carlos (June 28th 2021, 21/200-E). The patients/participants provided their written informed consent to participate in this study.

## Author contributions

All authors contributed to the study conception and design. Material preparation, data collection and analysis were performed by IP-F, IJ, AA, AL-G, RG-G. NR, BR-C, MM and BP-J. The first draft of the manuscript was written by IP-F, IJ, AA, EN and AS-F and all authors commented on previous versions of the manuscript. All authors contributed to the article and approved the submitted version.

## Supplementary material

Supplementary Figure 1 Individual trends of KTR patients. Levels of anti-Spike IgG antibody (BAU/mL) in KTR (black lines and circles) and HV (blue lines and squares) for each of the post-vaccination samples. Part of the KTR showed a delay in the generation of Spike-specific IgG at P1 and a higher decrease of IgG levels at P2 compared to controls.
