# Supplementary figures and images for: Role of mTOR inhibitor in the cellular and humoral immune response to a booster dose of SARS-CoV-2 mRNA-1273 vaccine in kidney transplant recipients
Source: Front Immunol. 2023 Feb 2;14:1111569. doi: 10.3389/fimmu.2023.1111569 (PMC9931894; doi:10.3389/fimmu.2023.1111569)

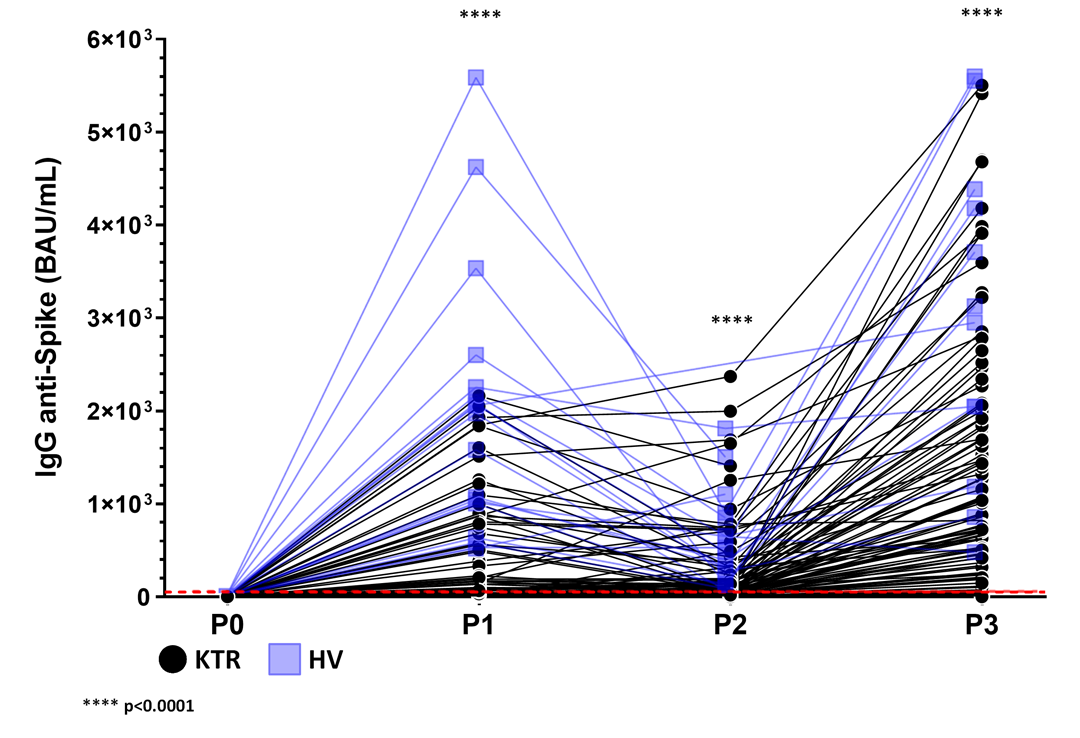

Supplement: Supplementary Figure 1 — Individual trends of KTR patients. Levels of anti-Spike IgG antibody (BAU/mL) in KTR (black lines and circles) and HV (blue lines and squares) for each of the post-vaccination samples. Part of the KTR showed a delay in the generation of Spike-specific IgG at P1 and a higher decrease of IgG levels at P2 compared to controls. [file Image_1.tif]
